# Endoscopic full thickness resection vs. transanal endoscopic microsurgery for local treatment of rectal neuroendocrine tumors - a retrospective analysis

**DOI:** 10.1007/s00384-020-03800-x

**Published:** 2020-11-19

**Authors:** Markus Brand, Stanislaus Reimer, Joachim Reibetanz, Sven Flemming, Marko Kornmann, Alexander Meining

**Affiliations:** 1 Medical Department II, Gastroenterology, University Hospital Wuerzburg, Oberduerrbacher Str. 6, Wuerzburg, 97080 Germany; 2 Department of General, Visceral, Transplantation, Vascular and Pediatric Surgery, University Hospital Wuerzburg, Wuerzburg, Germany; 3 Department of General and Visceral Surgery, University Hospital Ulm, Ulm, Germany

**Keywords:** Rectum, Neuroendocrine tumor (NET), Transanal endoscopic microsurgery (TEM), Endoscopic full thickness resection (eFTR), Full-thickness resection device (FTRD)

## Abstract

**Purpose:**

Local treatment of small well-differentiated rectal neuroendocrine tumors (NETs) is recommended by current guidelines. However, although several endoscopic methods have been established, the highest R0 rate is achieved by transanal endoscopic microsurgery (TEM). Since a recently published study about endoscopic full thickness resection (eFTR) showed a R0 resection rate of 100%, the aim of this study was to evaluate both methods (eFTR vs. TEM).

**Methods:**

We retrospectively analyzed all patients with rectal NET treated either by TEM (1999–2018) or eFTR (2016–2019) in two tertiary centers (University Hospital Wuerzburg and Ulm). We analyzed clinical, procedural, and histopathological outcomes in both groups.

**Results:**

Twenty-eight patients with rectal NET received local treatment (TEM: 13; eFTR: 15). Most tumors were at stage T1a and grade G1 or G2 (in the TEM group two G3 NETs were staged T2 after neoadjuvant chemotherapy). In both groups, similar outcomes for en bloc resection rate, R0 resection rate, tumor size, or specimen size were found. No procedural adverse events were noted. Mean procedure time in the TEM group was 48.9 min and 19.2 min in the eFTR group.

**Conclusion:**

eFTR is a convincing method for local treatment of small rectal NETs combining high safety and efficacy with short interventional time.

**Supplementary Information:**

The online version contains supplementary material available at 10.1007/s00384-020-03800-x.

## Introduction

Well differentiated neuroendocrine tumors (NETs) of the rectum are rare and often present as asymptomatic incidental findings during colonoscopy [1]. However, due to higher participation rates of patients to screening colonoscopies, these submucosal tumors are diagnosed more frequently [2, 3]. Most of those tumors are less than 10 mm in diameter and typically present as a small yellowish submucosal nodule often overlaid by a normal mucosal surface. Small rectal NETs (≤ 10 mm) present a low incidence of lymphovascular invasion and accordingly of metastasis. Thus, the current European Neuroendocrine Tumor Society (ENETS) guidelines recommend local treatment for G1/G2 rectal NETs below 10 mm in stage T1 and T2 and for G1/G2 rectal NET between 10 and 20 mm in stage T1 without lymph node metastasis (LNM) [1]. Radical surgical treatment is recommended in cases of G3 differentiation, T2 stage (10–20 mm), or size > 20 mm without LNM and distant metastasis [1]. Local treatment options include classical endoscopic methods (e.g. mucosectomy), advanced endoscopic techniques (endoscopic resection using an endoscopic variceal ligation device (EMR-L) or suction cap technique (EMR-C), endoscopic submucosal dissection (ESD), endoscopic full thickness resection (eFTR)), and transanal endoscopic microsurgery (TEM) with increased R0 resection rates according to advanced/invasive techniques [4–8]. A recent publication reported excellent R0 rates, using eFTR for rectal NET treatment. However, data comparing eFTR with other techniques is still missing [9]. In this study, we aim to analyze the outcome of eFTR and TEM in local treatment of rectal NETs.

## Materials and methods

We performed a retrospective analysis of all patients presenting with rectal NETs in two tertiary referral centers (University Hospital Wuerzburg and University Hospital Ulm), who received a local treatment either by TEM (between 1999 and 2018) or eFTR (between 2016 and 2019). All cases were primary cases without previous resection attempts. They were diagnosed by previous biopsy or referred to one of the centers for direct resection of the submucosal tumor. Procedural time, size of resected specimen, peri- and post-procedural adverse events, clinical outcomes, and histopathological results were retrospectively analyzed.

### eFTR procedure

**Fig. 1:**
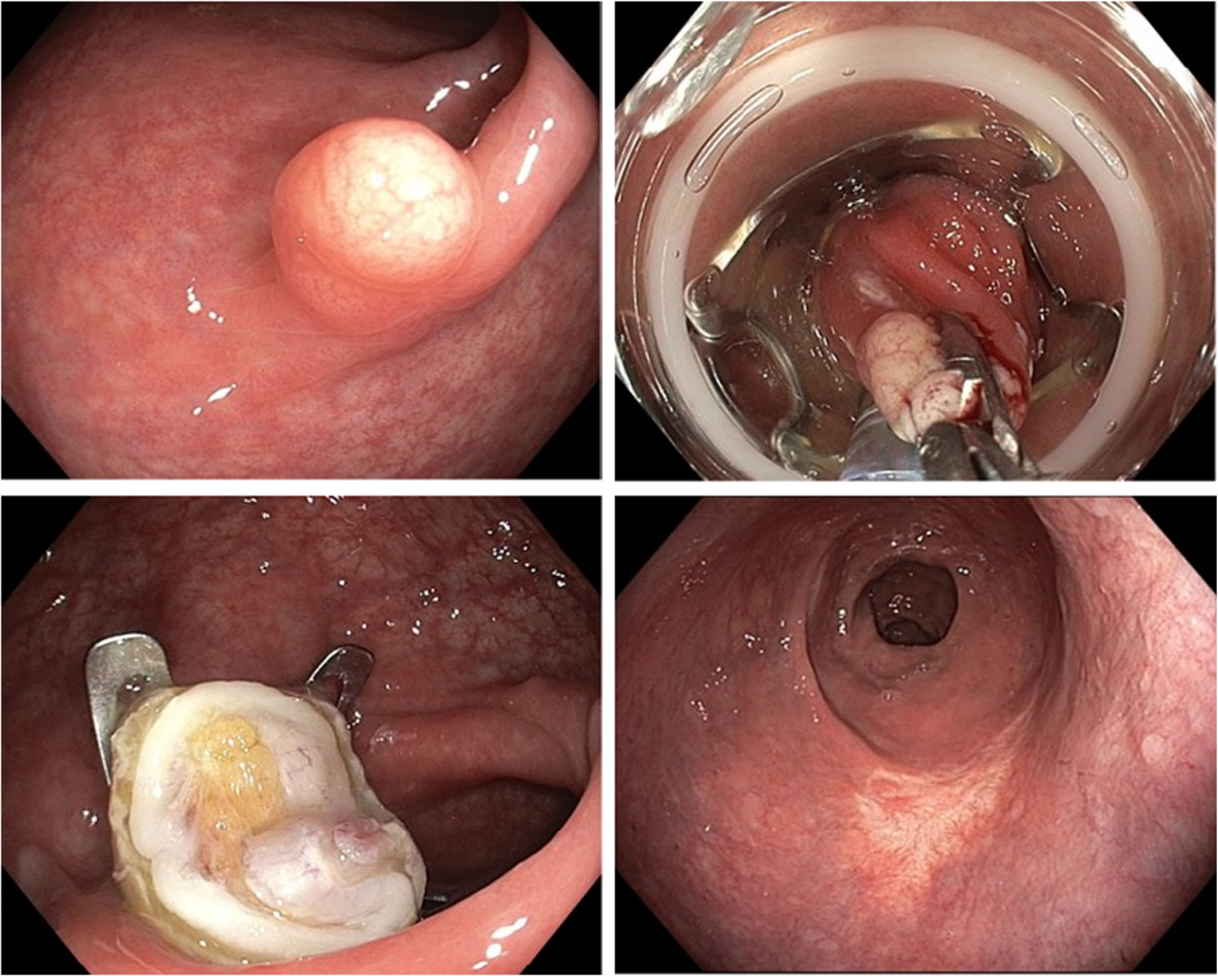
eFTR of rectal NET. **a R**ectal NET presenting typically as a small yellowish submucosal nodule with normal mucosal surface. **b P**ulling the NET into the FTRD by grasping forceps. **c R**esection site with OTSC in situ. **d R**esection scar 6 months after eFTR (OTSC had detached spontaneously). NET neuroendocrine tumor, FTRD full-thickness resection device, OTSC over-the-scope clip, eFTR endoscopic full thickness resection. (Numbering **a** upper left, **b** upper right, **c** lower left, **d** lower right)

eFTR was performed under nurse-administrated propofol sedation (NAPS). Standard colonoscopes (CF-H180 or CF-H190; Olympus Corp., Tokyo Japan) were used for the procedure. The full-thickness resection device (FTRD; Ovesco Endoscopy, Tuebingen, Germany) was attached at the tip of the endoscope and resection of rectal NET was performed as previously described (Fig. 1) [9, 10].

Resection time was defined as time from first introduction until last removal of endoscope as documented in the endoscopy procedure protocol.

### TEM procedure

Transanal microsurgery was performed in operating theater under general anesthesia using established technique and instruments [11]. Resection time was defined as the time from introducing until removing TEM instruments from the rectum. This time interval was determined from the operation protocol.

### Histopathological evaluation

Local pathology at each center evaluated the following characteristics: specimen size, tumor size, depth of tumor invasion, lateral and vertical margin involvement, lymphovascular invasion, Ki67-index, and tumor grading. En bloc resection was defined as removal of the tumor in a single piece. Complete resection (R0 resection) was defined as an en bloc resection without tumor cells visible at the lateral or vertical margins of the resected specimen. Complete full-thickness resection was defined as evidence of muscularis propria and perirectal adipose tissue in the resected specimens.

### Data collection

Patient characteristics, procedural adverse events, and clinical outcome were determined from patient records. Ethical approval for retrospective data analysis was given by the local ethics committee in the analyzing center (Wuerzburg). While size of resection specimen (length and width) was evaluated by the local pathologist, the area of the specimen (in cm^2^) was estimated using the formula for an ellipse (*A* = *a* × *b* × *π*).

### Statistical analysis

Statistical analysis of collected data was performed using Excel statistical software package (Microsoft Excel for Mac 2020). Results are presented as mean values. Due to the small group size, we present value range instead of standard deviation. Because of the retrospective character of the study and potential biases (long time range in TEM group, different treating physicians, no matched groups), we decided against a direct statistical comparison of both methods; thus, no *p* values were calculated.

## Results

In this retrospective analysis, we found 28 patients with rectal NET who received local treatment either by TEM (13 patients between 1999 and 2018) or eFTR (15 patients between 2016 and 2019). Patient’s characteristics are summarized in Table 1.

**Table 1:** Patients characteristics

	TEM	eFTR
Characteristic	13	15*
Sex, men/women	6/7	11/4
Age, mean (range)	53.0 (31–77)	53.9 (19–80)
Tumor localization, mean cm from anal verge (range)	4.7 (1–10)	7.3 (3–11)
Tumor size, mean mm (range)	6.7 (1–17)	4.6 (2–8)

*TEM* transanal endoscopic microsurgery, *eFTR* endoscopic full thickness resection

*In one eFTR specimen, no further tumor cells were found after initial biopsy

In the TEM group, the mean tumor size was 6.7 mm with a range from 1 up to 17 mm. The mean localization of the rectal NET (cm from anal verge) was 4.7 cm, with five NETs located close to the anal verge (0.5 to 1.0 cm). In the eFTR group, all NETs were smaller than 10 mm (mean 4.6 mm), and mean localization was 7.4 cm from anal verge. Minimal distance to anal verge in the eFTR group was 3.0 cm. In one eFRT procedure, no residual tumor cells were found in the specimen after initial biopsies - thus, the rectal NET was already completely removed by biopsies. En bloc resection was achieved in 92% (12/13) in the TEM group and in 100% (15/15) in the eFTR group. The R0 resection rate was 92% in the TEM group and 100% in the eFTR group. Due to R1 situation, one patient in the TEM group underwent surgical therapy (lower anterior resection (LAR) 2 months after local treatment. The mean procedure time in the TEM group was 48.9 min and the mean size of the resected specimen was 2.9 cm^2^. In 12 of 13 cases (92%), TEM resulted in complete full-thickness wall resection. In the eFTR group, the mean procedure time was 19.2 min. Complete full-thickness wall resection was achieved in 6 of 15 eFTR procedures (40%); mean size of the resected specimen was 2.5 cm^2^. All NETs in the eFTR group were staged T1a and either grade G1 or G2. In the TEM group, two G3 NETs were resected after neoadjuvant chemotherapy. Those tumors were at stage T2. The other NETs in the TEM group were stage T1a and grade G1 or G2. Histopathological and procedural results are shown in Table 2.

**Table 2:** Histopathological and procedural results

	TEM	eFTR
Characteristic	13	15*
En bloc resection, yes/no	12/1	15/0
R0 resection, yes/no	12/1	14/0
Complete FTR, yes/no	12/1	6/9
Size of resection specimen, mean cm^2^ (range)	2.9 (0.2–8.8)	2.4 (0.9–6.3)
Procedure time, mean min (range)	48.9 (20–117)	19.2 (11–26)
Tumor stage, T1a/T2	11/2	14/0
Tumor grading, G1/G2/G3	10/1/2	10/4/0
Lymphovascular invasion, yes/no	0/13	0/14
Venous invasion, yes/no	1/10	0/14
Adverse events, yes/no	0/13	0/15

*TEM* transanal endoscopic microsurgery, *eFTR* endoscopic full thickness resection, *R0* residual 0, *G1* grade 1, *G2* grade 2, *G3* grade 3

*In one eFTR specimen, no further tumor cells were found after initial biopsy

Both patients with G3-NET were treated as an individual approach decided by the interdisciplinary tumor board review. The first patient (female, 44 years old) had a G3 rectal NET and limited liver metastasis at primary diagnosis. She refused LAR with lymph node resection. Therefore, she received neoadjuvant chemotherapy in a two-step approach (TEM for local resection followed by liver surgery). Unfortunately, diffuse metastasis occurred 3 months later during adjuvant chemotherapy. The second patient (male, 54 years old) also refused LAR - initially he refused even TEM. After intensive discussion, he agreed in local resection by TEM after neoadjuvant chemotherapy

In both groups, no peri- or post-interventional adverse events occurred. In nine patients, data of local follow-up after eFTR (after 6–8 months) was available - in all cases, the over-the-scope clip (OTSC) had detached spontaneously as seen on follow-up examinations. Detailed information about every individual case is provided in the Supplementary Material.

## Discussion

Neuroendocrine tumors account for about 1% of all cancers in the gastrointestinal tract; however, due to improvement in diagnostic techniques, they are diagnosed more frequently [12]. The same holds true for rectal NETs that account for about 1.3% of off all rectal tumors [13]. Well-differentiated NETs frequently remain asymptomatic; thus, they often present as incidental findings during cancer screening colonoscopies [2, 3]. Prognosis of G1 rectal NETs with a size of < 10 mm is excellent and the 5-year survival rate is almost 100% [2, 14, 15]. Thus, guidelines suggest that small rectal NETs without risk factors (limited to submucosal layer, maximal diameter 10 mm, grading G1, no lymphovascular or venous invasion) should be treated locally [1, 12]. Two studies published in 2019 showed that rectal NETs > 10 mm have a low, but not negligible, risk of LNM, while this risk is minimal in tumors ≤ 10 mm [15, 16]. Data about the risk of lymph node metastasis depending on tumor grading (especially in grade G2) are still poor; thus, no clear cutoff value for local treatment has yet been established. The current ENETS guideline published in 2016 recommends local treatment for rectal NET G2 between 10 and 20 mm without LNM, followed by radical surgery, if local resection is incomplete [1]. Therefore, the optimal local treatment procedure should combine highest local R0 rate with lowest complication rate. With increasing incidence of well-differentiated rectal NETs, several studies investigated endoscopic resection modalities to find the optimal method for those submucosal tumors. In most of those studies, the conventional EMR presents poor R0 rates ranging from about 40 to 80% [4, 5, 8]. Thus, advanced EMR techniques have been evaluated to improve local R0 rate. The most frequently used techniques are EMR-C and EMR-L, with R0 rates between 80 and 100% [4–6, 17]. Another established resection technique for rectal NETs is ESD; however, this method is challenging, more complicated, and especially in western countries limited to few specialized centers. Therefore, it is not surprising that R0 resection rate in recent Asian studies is about 90 to 100% while the only European study found only about 80% [4–6]. The main reason for incomplete resection of rectal NETs are remaining tumor cells in the deeper part of the submucosal layer; therefore, histopathological R1 situation sometimes occurs at the vertical margin of resected specimens, even if EMR-L/C or ESD technique is used [4]. Hence, eFTR might help to overcome this problem. Studies using TEM for treatment of rectal NETs achieved 100% R0 rate [18–20]. However, TEM is very time consuming (mean time in literature varies from 45 to 80 min), cost expensive, and limited to rectal NETs located in the lower two-thirds of the rectum [11]. EFTR closes the gap between TEM and advanced endoscopic technique by combining the advantages of both procedures. To the best of our knowledge, the first data about eFTR in rectal NETs (subgroup analysis from a multicenter study with 31 centers using FTRD) identified 40 eFTRs (28 NETs, 12 granulation tissues) with a R0 rate of 100% [9]. Our study is the first study that analyzes eFTR and TEM in two tertiary centers. We found 100% R0 rate in the eFTR group as well, and the mean resection time was comparable too (19.2 min vs. 18.5 min in the multicenter study). In our TEM group, the R0 rate was 92% compared to 100% in other studies, while resection time (48.9 min) is similar as previously described [11].

However, there are some limitations about the eFTR system. In contrast to TEM, there is a limitation in maximal resectable tumor size due to the diameter of the FTRD (primary study demonstrated good technical efficacy in lesions ≤ 20 mm); however, this is not a strong limitation as rectal NETs > 20 mm should undergo surgical resection [1, 10, 12]. Another potential limitation of eFTR in the rectum is that resection of the muscularis propria is sometimes incomplete. In our study, incomplete FTR occurred in 60% compared to 5% in the multicenter study; however, we used the evidence of perirectal adipose tissue in the resected specimens to determine FTR, while the multicenter study did not. Nevertheless, in our study, the eFTR group achieved 100% R0 resections, because all rectal NETs were in stage T1a, at which complete resection of the submucosal layer is enough for local treatment. Moreover, in cases of incomplete NET resection by eFTR, a second procedure or additional TEM as a rescue procedure is still possible. Furthermore, the use of the eFTRD in lesions close to the anal verge is sometimes technically difficult. This could explain why we found the five cases in the TEM group with NET close to the anal verge (0.5 to 10 mm), while no such case was found in the eFTR group.

Limitations of the study are its retrospective nature; therefore, we mainly focused on procedural and histopathological results that were available for all patients. Due to low incidence of rectal NETs, patient numbers in both groups are relatively small. Moreover, the time range of the cases in the TEM group was 20 years (1999–2018) while eFTR patients were treated in 4 years (2016–2019). This is due to increasing numbers of rectal NET patients in our centers in the last 5 years and the availability of the FTRD system since 2016. Since histopathological methods in NETs developed significantly during the last 20 years, the comparison of resected specimens over a period of approximately 20 years is somewhat limited.

In conclusion, eFTR appears to be an effective resection method for well-differentiated rectal NET of smaller size, combining high R0 resection rate with low complication rate and short interventional time.

## Supplementary Information

ESM 1 (DOCX 13 kb).

ESM 2 (DOCX 13 kb).
