# Supplementary material for: Endoscopic full thickness resection vs. transanal endoscopic microsurgery for local treatment of rectal neuroendocrine tumors - a retrospective analysis
Source: Int J Colorectal Dis. 2020 Nov 19;36(5):971–6. doi: 10.1007/s00384-020-03800-x (PMC8026435; doi:10.1007/s00384-020-03800-x)
Supplement: Supplementary file 1 — (DOCX 13 kb). [file 384_2020_3800_MOESM1_ESM.docx]

**Supplementary material I**

| Case year | Age | Sex | Distance to anal verge (cm) | Specimen size (cm²) | Tumorsize (mm) |
| --- | --- | --- | --- | --- | --- |
| 2016 | 40 | w | 10 | 1,53 | 4 |
| 2016 | 59 | m | 3 | 2,36 | 5 |
| 2016 | 61 | m | 10 | 1,84 | 8 |
| 2016 | 65 | w | 7 | 2,94 | 8 |
| 2017 | 56 | m | 5 | 2,00 | 2 |
| 2017 | 52 | m | 4 | 1,89 | 2 |
| 2017 | 49 | m | 5 | 1,60 | * |
| 2018 | 19 | m | 11 | 1,76 | 5 |
| 2018 | 37 | w | 10 | 0,85 | 2 |
| 2018 | 69 | m | 7 | 1,42 | 5 |
| 2018 | 80 | m | 5 | 1,26 | 5 |
| 2019 | 48 | m | 10 | 2,26 | 3 |
| 2019 | 58 | m | 7 | 6,28 | 6 |
| 2019 | 57 | m | 8 | 1,90 | 2 |
| 2019 | 59 | w | 7 | 5,42 | 8 |

**Table 1** Detailed information about the 15 eFTR cases

eFTR endoscopic full thickness resection, m: men, w: women, * in one eFTR specimen no further tumor cells were found after initial biopsy
