# Supplementary material for: Endoscopic full thickness resection vs. transanal endoscopic microsurgery for local treatment of rectal neuroendocrine tumors - a retrospective analysis
Source: Int J Colorectal Dis. 2020 Nov 19;36(5):971–6. doi: 10.1007/s00384-020-03800-x (PMC8026435; doi:10.1007/s00384-020-03800-x)
Supplement: Supplementary file 2 — (DOCX 13 kb). [file 384_2020_3800_MOESM2_ESM.docx]

**Supplementary material II**

| Case year | Age | Sex | Distance to anal verge (cm) | Specimen size (cm²) | Tumorsize (mm) |
| --- | --- | --- | --- | --- | --- |
| 1999 | 52 | w | 7 | 0,2 | 1 |
| 2000 | 54 | m | 8 | 5,3 | 4 |
| 2004 | 49 | m | 8 | 2,2 | 6 |
| 2009 | 77 | w | 10 | 1,56 | 5 |
| 2010 | 54 | m | 1 | 3,54 | 10 |
| 2010 | 54 | m | 1 | 3,54 | 10 |
| 2010 | 40 | w | 6 | 1,41 | 5 |
| 2012 | 31 | w | 5 | 3,43 | 4 |
| 2014 | 51 | m | 4 | 1,18 | 1,5 |
| 2017 | 73 | w | 8 | 1,65 | 8 |
| 2017 | 44 | w | 1 | 8,84 | 17 |
| 2018 | 56 | m | 1 | 1,77 | 1,5 |
| 2018 | 54 | w | 0.5 | 3,34 | 14 |

**Table 2** Detailed information about the 13 TEM cases

TEM transanal endoscopic microsurgery, m: men, w: women
